# Comparison of two electronic hand hygiene monitoring systems in promoting hand hygiene of healthcare workers in the intensive care unit

**DOI:** 10.1186/s12879-020-05748-3

**Published:** 2021-01-11

**Authors:** Xiao Zhong, Dong-Li Wang, Li-Hua Xiao, Lan-Fang Mo, Qing-Fei Wu, Yan-Wei Chen, Xiao-Feng Luo

**Affiliations:** 1 Department of Nosocomial Infection Control, Shenzhen Hospital, University of Chinese Academy of Sciences, Shenzhen, 518106 Guangdong China; 2 Inspection center, Guangming District Center for Disease Control and Prevention, Shenzhen, Guangdong China

**Keywords:** Hand hygiene, Electronic hand hygiene monitoring systems, Interrupted time series analyse, Infection prevent and control, Reminder and feedback

## Abstract

**Background:**

Hand hygiene (HH) is the cornerstone of infection control, and the promotion of HH is the focus of the world. The study aims to compare the role of two different types of electronic hand hygiene monitoring systems (EHHMSs) in promoting HH of healthcare workers (HCWs) in the intensive care unit (ICU).

**Methods:**

In a 16-bed ICU of a general tertiary hospital in Shenzhen, the research was divided into three stages with interrupted time series (ITS) design. In the first stage, the direct observation method was used to monitor and feed back the HH compliance rate of HCWs monthly. In the second stage, the type1 EHHMS was applied to monitor and feed back the individual number of HH events monthly. In the third stage, the type2 EHHMS with a function of instant reminder and feedback was employed, and the personal HH compliance rates were fed back monthly. Meanwhile, direct observation continued in the last two stages.

**Results:**

In the second stage, The HH compliance rate increased. However, there was no significant difference in the trajectory of the rate compared with the first stage. In the first month of the third stage, the HH compliance rate increased by 12.324% immediately and then ascended by 1.242% over time. The number of HH events per bed day and HH products’ consumption per bed day were consistent with the change of HH compliance rate observed.

**Conclusion:**

Monitoring and feedback can improve the HH of HCWs. The EHHMS, with the function of real-time reminders and feedback, has a more noticeable effect on promoting HH.

**Supplementary Information:**

The online version contains supplementary material available at 10.1186/s12879-020-05748-3.

## Background

Hand hygiene is currently recognized as the cornerstone of healthcare-associated infection (HAI) control [1]. It can effectively prevent the hands from becoming the vectors of HAIs pathogens, consequently reducing the occurrence of HAIs [2–5]. In 2009 the WHO issued five critical HH moments [4]: 1. Before contact with the patient; 2. Before cleaning and aseptic operation; 3. After a body fluid exposure risk; 4. After contact with the patient; 5. After touching the patient’s surroundings. These five HH moments are the principles that every HCW should follow in their work. However, the low HH compliance of HCWs has been a global problem [6]. The overall median HH compliance rate was 40%, and the HH compliance rates were lower in ICU (30–40%) than in other settings (50–60%), which was reported by a systematic review contained 96 studies [7]. The low HH compliance rate was associated with sustained work stress faced by HCWs in the complex modern healthcare environment [8]. Therefore, HH’s promotion among HCWs is a challenge and focus for different levels and kinds of medical establishments.

Monitoring & feedback is the most commonly used measure to promote HH among HCWs, compared with training, education, and reminders [9], which have proved to be one of the useful measures among the multimodal measures for HH promotion [10]. At present, direct observation is still the golden standard of HH monitoring. Even though it has many defects [11, 12]: time and labor consuming, an insufficient number of hand hygiene events (HHEs) observed, Hawthorne effect, and observer bias [4, 13]. To solve or reduce the direct observation method’s shortcomings, a new HH monitoring tool, namely the EHHMS, has been gradually developed in recent years. The EHHMS can be defined as the auxiliary system of direct observation or the automatic electronic system that can continuously collect data such as HH moments, HHEs, or HH product consumption. It can count, analyze, and feed back the data. Such as dispenser and handwashing counting systems, Wi-Fi identity badges modified to detect alcohol vapors, radiofrequency identification badge systems, and automated HH monitoring networks [14–16].

Some infection control experts have reported the applications of EHHMSs to promote HH of HCWs. For example, Edmisten C et al. [12] applied EHHMS in three community hospitals to monitor and improve the HH of HCWs to keep the HH compliance rate above 85%. Pong S et al. [17] used the EHHMS with a reminder function to monitor the HH of HCWs entering and leaving the wards and found that the number of HHEs doubled after the adoption of the system, and it decreased by 0.18% per week after the removal of the system. However, different kinds of EHHMSs may play different roles in the promotion of HCWs’ HH. Recently, few articles have compared the effects of different EHHMSs in promoting HH among HCWs. Therefore, it was necessary to explore what kinds of EHHMSs could better promote the HH of HCWs.

## Methods

### Study object and design

This study was carried out in a general ICU of a tertiary hospital with 1350 open beds in Guangming District, Shenzhen city, Guangdong province, China. The ICU has 16 beds and 46 HCWs, including 11 doctors and 35 nurses. The mean ages of HCWs were 32.5 ± 6.8 years. Five of the 35 nurses were male, and all the doctors were male. This study was a quality improvement activity and had been approved by the hospital ethics committee. All HCWs in the ICU were selected as study subjects, and informed consent was signed. Those who resigned or transferred to another department would automatically withdraw from the study, and recruits would need to be retrained and sign informed consent.

To compare the effects of two kinds of EHHMSs, from March 2018 to December 2019, our hospital successively used two different EHHMSs to monitor the HH of HCWs in the ICU. Meanwhile, the direct observation method was used to evaluate the effect of two EHHMSs. The study adopted the method of ITS design, which was divided into three stages. In the first stage: from March 2017 to February 2018, direct observation was used to monitor the HH of ICU HCWs according to WHO’s five HH moments. The total HH compliance rate was reported to the department monthly. In the second stage: from March 2018 to January 2019, the type1 EHHMS was used to monitor the HH of ICU HCWs. The system can track the HHEs and the consumption of alcohol-based hand rubs (ABHRs) & liquid soaps but does not recognize HH moments. The ranking of individual HHEs per bed day was fed back to the department every month. In the third stage: from February 2019 to December 2019, the type2 EHHMS was used to monitor the HH of ICU HCWs. The system can automatically identify the HH indicators, including before and after contacting the patient, after touching the patient’s surrounding environment and objects, and immediately reminded the HCWs to perform HH. The personal HH compliance rate ranking was fed back to the department monthly. In the second and third stages, direct observation continued to monitor the HH of HCWs. For exploring the effects of two EHHMSs on HH of ICU HCWs, the variation trend of HCW’s HH compliance rate, the HHEs, and the consumption of ABHRs & liquid soaps were compared. During these three stages, all other infection control measures remained unchanged, nor did any other form of HH promotion movement.

### The direct observational method

Two observers, composed of infection control professionals, with more than 10 years of work experience and an average age of 35 years, had been trained and educated repeatedly according to the principles of “My five moments for hand hygiene” in advance. Kappa statistics had been performed to ensure that their inter-observer compliance reached over 75%. Then a detailed observation schedule that specified sessions for observation was formulated. Each session lasted 30 min, including both busy and free periods. The two observers were randomly assigned to observe four busy and free periods each month for 4 h. From March 2017 to December 2019, the two observers used the mobile phone APP (HH observation auxiliary system) to perform the observations according to the observation scheme. The APP could reduce the Hawthorne effect compared with the conventional paper observation table [16]. Observers in ICU wards used this APP instead of paper observation forms to observe and record the HH of HCWs, which was relatively hidden and not easily detected. When the HCWs noticed the observers’ presence, they assumed the observers were looking at the phone. Because the observers were fixed for a long time, it was hard to avoid detection. When in doubt, observers explained that they were examining other items. Through this unobtrusive observation, the Hawthorne effect brought by the observers was avoided as much as possible. The total HH compliance rate was fed back to the HCWs every month.

### The type1 EHHMS

**Fig. 1:**
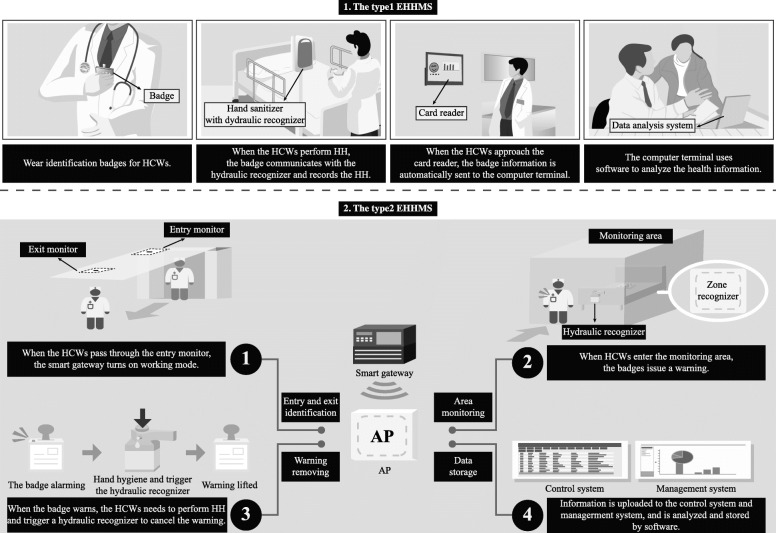
The function flow chart of type1 and type2 EHHMS. Note. EHHMS, electronic hand hygiene monitoring system; HH, hand hygiene; HCW, healthcare worker; AP, cloud access point

From March 2018 to January 2019, the type1 EHHMS was used to monitor the HH of ICU HCWs. An additional file shows the technical parameters of type1 EHHMS (Additional file 1: The technical parameters. Part 1.). The system is a monitoring system for HHEs of HCWs. ABHRs or liquid soap dispensers with sensors were installed near every ICU bed and sink, and identification badges were worn on the chest of HCWs. When HCWs placed their hands on the dispensers’ outlet, the outlets would spray the liquid automatically, and the badges would receive a signal from the sensor to record the HHEs. The sensor had a 10-s locking function, and if the same HCW took the liquid several times in 10 s, only one HHE would be recorded. The information recorded by the identification badges was inputted into the computer through the card reader daily to obtain the HHEs of the HCWs automatically. Simultaneously, the system could get the consumption of ABHRs & liquid soaps by multiplying the number of spraying of the dispensers by the amount of each ejection (2 ml). The ranking of individual HHEs per bed day was fed back to the HCWs every month. As shown in Fig. 1.

### The type2 EHHMS

From February 2019 to December 2019, the type2 EHHMS was used to monitor the HH of ICU HCWs. An additional file shows the technical parameters of type2 EHHMS (Additional file 1: The technical parameters. Part 2.). The system is an automated HH monitoring network. The bed zone recognizers were installed in each ICU bed. The dispensers of ABHRs or liquid soap with the bottle recognizers were installed near patient beds or sinks, and identity badges were worn for ICU HCWs. When the HCWs approached the bed zone, the badges would communicate with the bed zone recognizers. After 5 s, the badges would light up, and a short beep would remind the HH three times. The HCWs needed to carry out HH and trigger the hydraulic recognizer to remove the alarm. If no HH after 20 s of reminding, the badge longly beeped, the red light went out, and the system recorded a no HHE. If there was no HH after leaving the bed, the badge’s red glow flashed, and a short beep reminded the HH after the 160 s. After the reminder, there was still no HH within 25 s, and the system recorded it as no HHE. All data were automatically transmitted to the cloud access point (AP) installed on the ward ceiling, which uploaded the data to the background management system for storage and statistical analysis. Similar to the previous EHHMS, the system also recorded the consumption of ABHRs & liquid soaps. The ranking of individual HH compliance rate was fed back to the HCWs monthly. As shown in Fig. 1.

### Monitoring indicators

1. HHEs per bed day = the actual number of HHEs / the actual total number of occupied bed days. The HHEs mean the times or number of HH actions. The ICU nurses recorded the actual number of occupied bed days at 12 o’clock each night, including the actual occupied temporary beds. Patients who died or were discharged before 12 o’clock in the evening after admission should also be counted.
2. HH compliance rate = the total number of actual HHEs/ the total number of HH moments × 100%.
3. Consumption of ABHRs & liquid soaps per bed day = the total consumption of ABHRs & liquid soaps/actual total number of occupied bed days.

### Data analysis

The data were inputted into Excel 2016 to establish a database. The software Stata 15.1 (Stata Corporation, College Station, TX, US) was used for descriptive analysis, correlation analysis, and ITS analysis. First, an actest was performed to verify whether there was autocorrelation of time series data and determined the maximum lag levels. Then monthly data were analyzed by the ITSA package in Stata software. The Newey-west model of ITSA was selected for analysis with the lags determined previously. This model provides Newey-west standard error to solve autocorrelation and possible heteroscedasticity of data based on the ordinary least-squares regression. There were no control groups due to data limitations, and a single time series analysis model was used. The form of the model is as follows:

$$ \mathrm{Y}\_\mathrm{t}=\mathrm{Beta}\_0+\mathrm{Beta}\_1\left(\mathrm{T}\right)+\mathrm{Beta}\_2\left(\mathrm{X}\_\mathrm{t}\right)+\mathrm{Beta}\_3\left(\mathrm{T}\mathrm{X}\_\mathrm{t}\right) $$

Y_t is the result variable measured at each interval point t. T is the time since the study begins, X_t is the dummy variable representing the intervention (0 before the intervention, 1 otherwise), and TX_t is the interaction term. Beta_0 represents the intercept of the outcome variable. Beta_1 is the slope of the outcome variable before the intervention. Beta_2 represents the change in the outcome variable’s level immediately after the introduction of the intervention (compared to the counterfactual case). Beta_3 represents the difference in outcome variable slope before and after the intervention. Therefore, the significant *p* values in Beta_2 indicated immediate intervention effects, or intervention effects over time in Beta_3.

The continuous variables accord with normal distribution were present as the means±standard deviation (SD). Frequencies and percentages described the categorical variables, and the comparisons between the groups were conducted by a chi-square test or Fisher’s exact probability method. Statistical significance was observed at an α level of 0.05.

## Results

From March 2017 to December 2019, there were 1787 inpatients in the ICU, and the length of stay was 7138 days. There were 46 HCWs in the ICU. To maintain the ICU’s workload, the number of HCWs remained stable, even if three nurses and one doctor changed during the study period. A total of 6253 times of HH moments and 4377 times of HHEs were observed by direct observation, and the total HH compliance rate was 70.00%. The HHEs detected by EHHMS were 425,602 times, 59.37 times per bed day. The total consumption of ABHRs & liquid soaps was 847,137 ml, 118.2 ml per bed day.

**Fig. 2:**
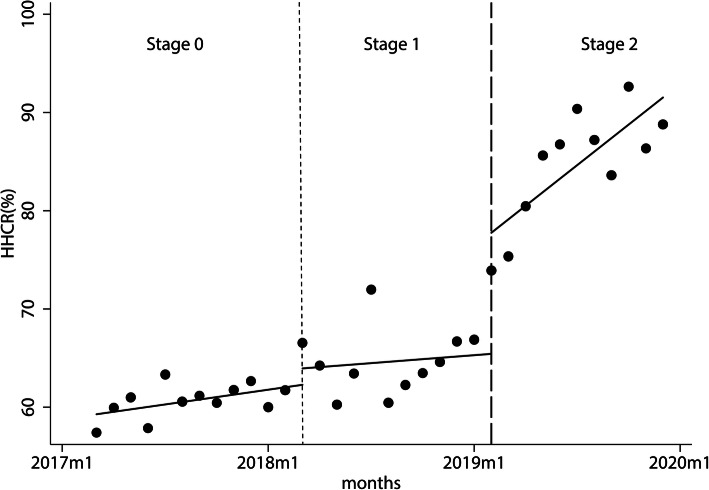
The variation of hand hygiene compliance rate in the three stages obtained by the direct observation method. The monthly HHCR from Mar. 2017 to Dec. 2019 obtained by the direct observation method. The black spots show the actual HHCR, and the full line shows the predicted trend of the HHCR. The short dash vertical line represents the introduction of the type1 EHHMS (Mar. 2018), and the long dash vertical line depicts the application of the type2 EHHMS (Feb. 2019). The two vertical lines divide the time into three stages, which are stage 0, stage 1, and stage 2. Note. HHCR, hand hygiene compliance rate; m, month

According to the actest test, the maximum lag order was 1 for the HH compliance rate, the number of HHEs per bed day, and HH products’ consumption per bed day. Therefore, the Newey-west model, with the lag order of 1, was used to analyze the time-series data. The results showed that the HH compliance rate in the first stage increased by 0.250% per month. In March 2018, the first month of the use of the type1 EHHMS, the HH compliance rate of HCWs increased by 1.667%. It then fell at a rate of 0.115% per month (relative to the first stage), but the difference was not statistically significant. In the first month using the type2 system, the HH compliance rate increased immediately by 12.324% and then increased at a rate of 1.242% per month relative to the second stage, as shown in Table 1 and Fig. 2.

**Table 1:** The variation of hand hygiene compliance rate in the three stages obtained by the direct observation method

HHCR	Coef.	Ne Wey-West Std. Err.	*t*	*P* > *|t|*	[95% CI]	
*_t*	.250	.1060	2.36	0.026	.033	.467
_x2018m3	1.667	1.885	0.88	0.384	−2.194	5.528
_x_*t*2018m3	−.115	.254	−0.45	0.654	−.635	.405
_x2019m2	12.324	3.152	3.91	0.001	5.867	18.780
_x_*t*2019m2	1.242	.440	2.82	0.009	.341	2.144
_cons	59.281	.742	79.89	0.000	57.761	60.801

Note. *HHCR* hand hygiene compliance rate, *Coef* coefficient, *Std. Err.* standard error, *CI* confidence interval, _*t* time since the start of the study, *m* month, *_x(trperiod)* dummy variable which representing the intervention periods (preintervention periods 0, otherwise 1), *_x_t(trperiod)* the interaction of _x and _*t*; _cons, constant

**Fig. 3:**
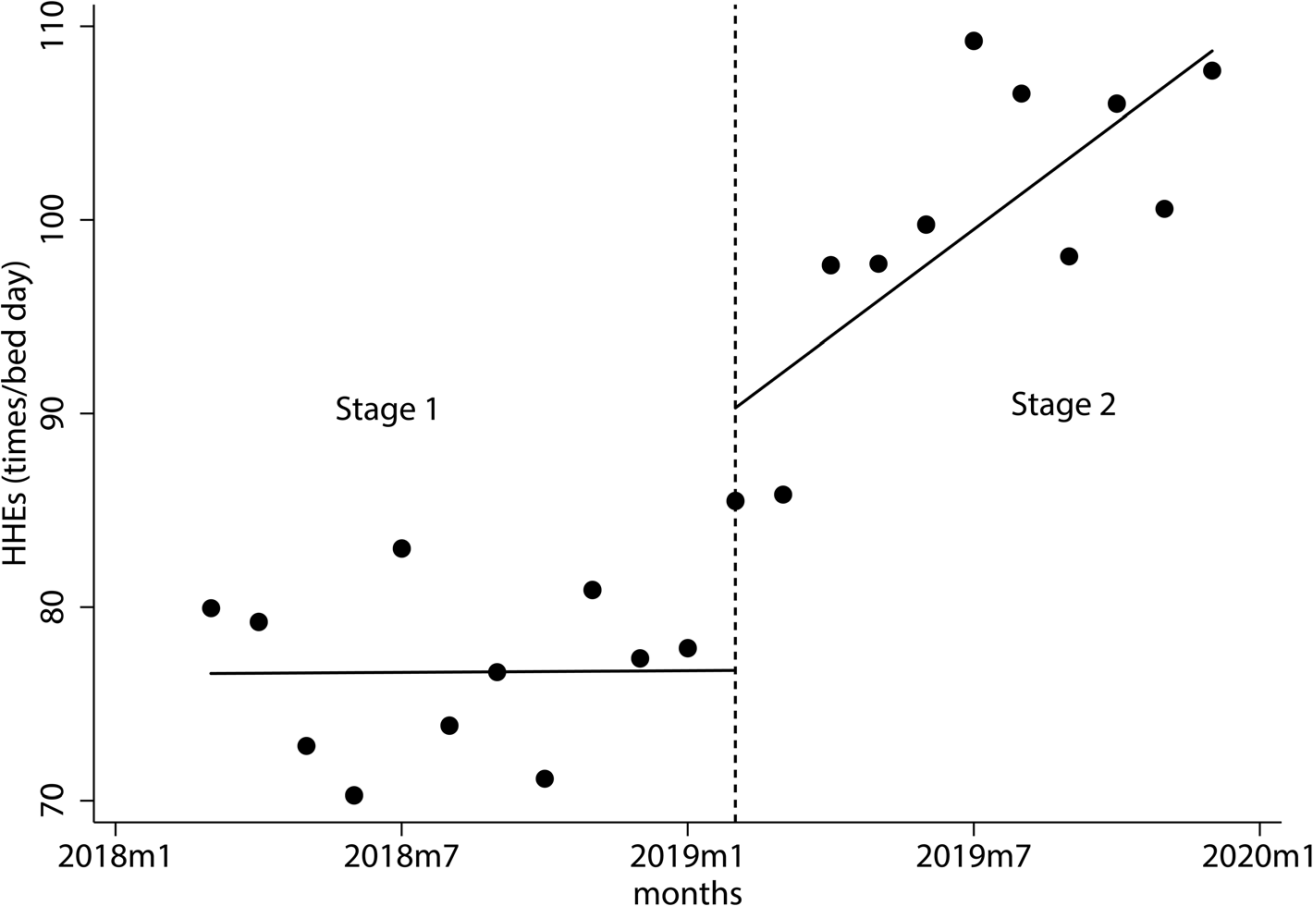
The variation of hand hygiene events per bed day in stages 1 and 2. The monthly HHEs per bed day from Mar. 2018 to Dec. 2019 obtained by EHHMS. The black spots show the actual HHEs per bed day, and the full line shows the predicted trend of the HHEs per bed day. The short dash vertical line depicts the application of the type2 EHHMS (Feb. 2019). The vertical lines divide the time into two stages, which are stage 1 and stage 2. Note. HHEs, hand hygiene events; m, month

**Fig. 4:**
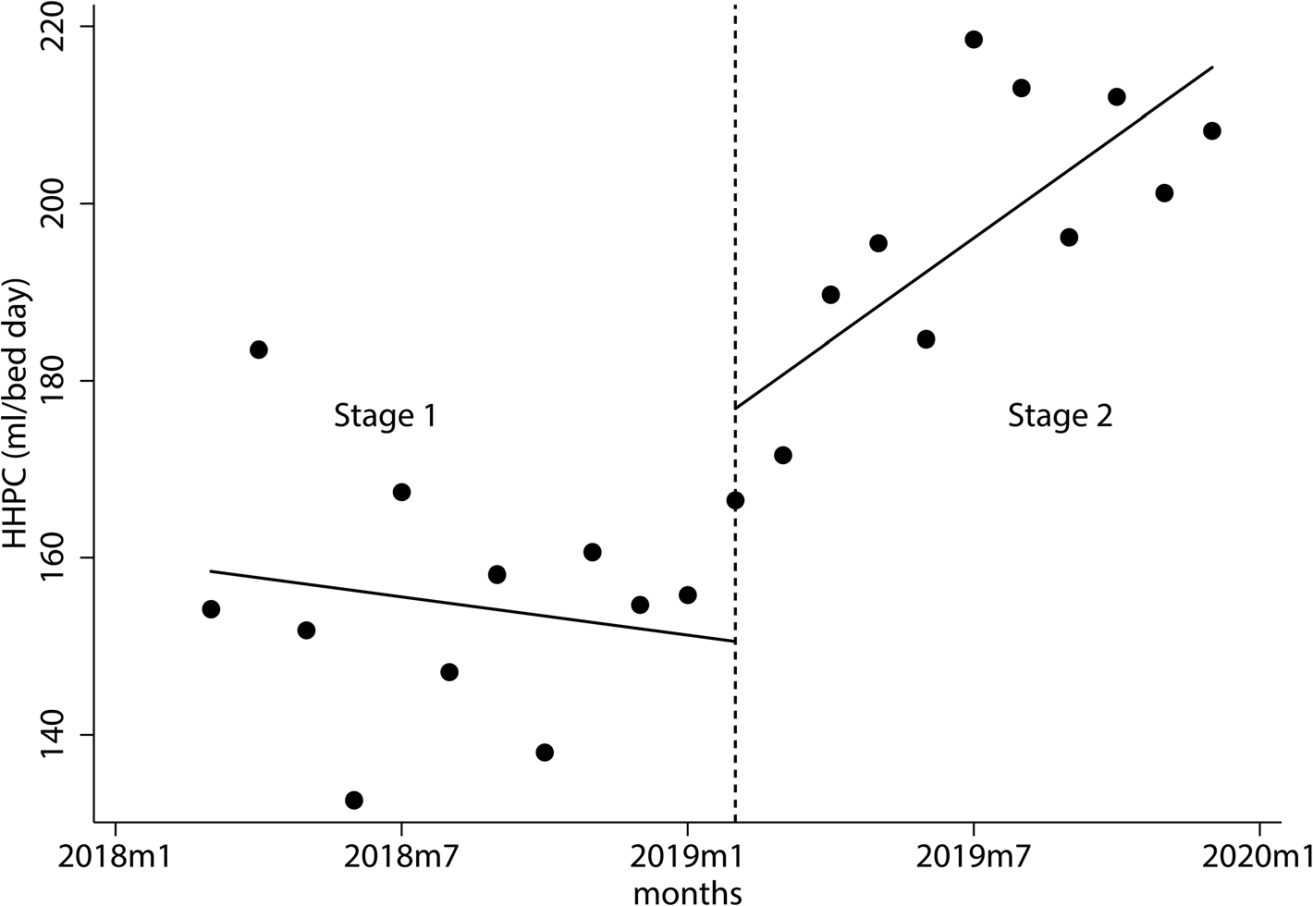
Changes in consumption of ABHR and liquid soap per bed day in stages 1 and 2. The monthly hand hygiene product consumption from Mar. 2018 to Dec. 2019 obtained by EHHMS. The black spots show the actual hand hygiene product consumption, and the full line shows the predicted trend of the hand hygiene product consumption. The short dash vertical line depicts the application of the type2 EHHMS (Feb. 2019). The vertical lines divide the time into two stages, which are stage 1 and stage 2. Note. HHPC, hand hygiene product consumption; m, month

**Fig. 5:**
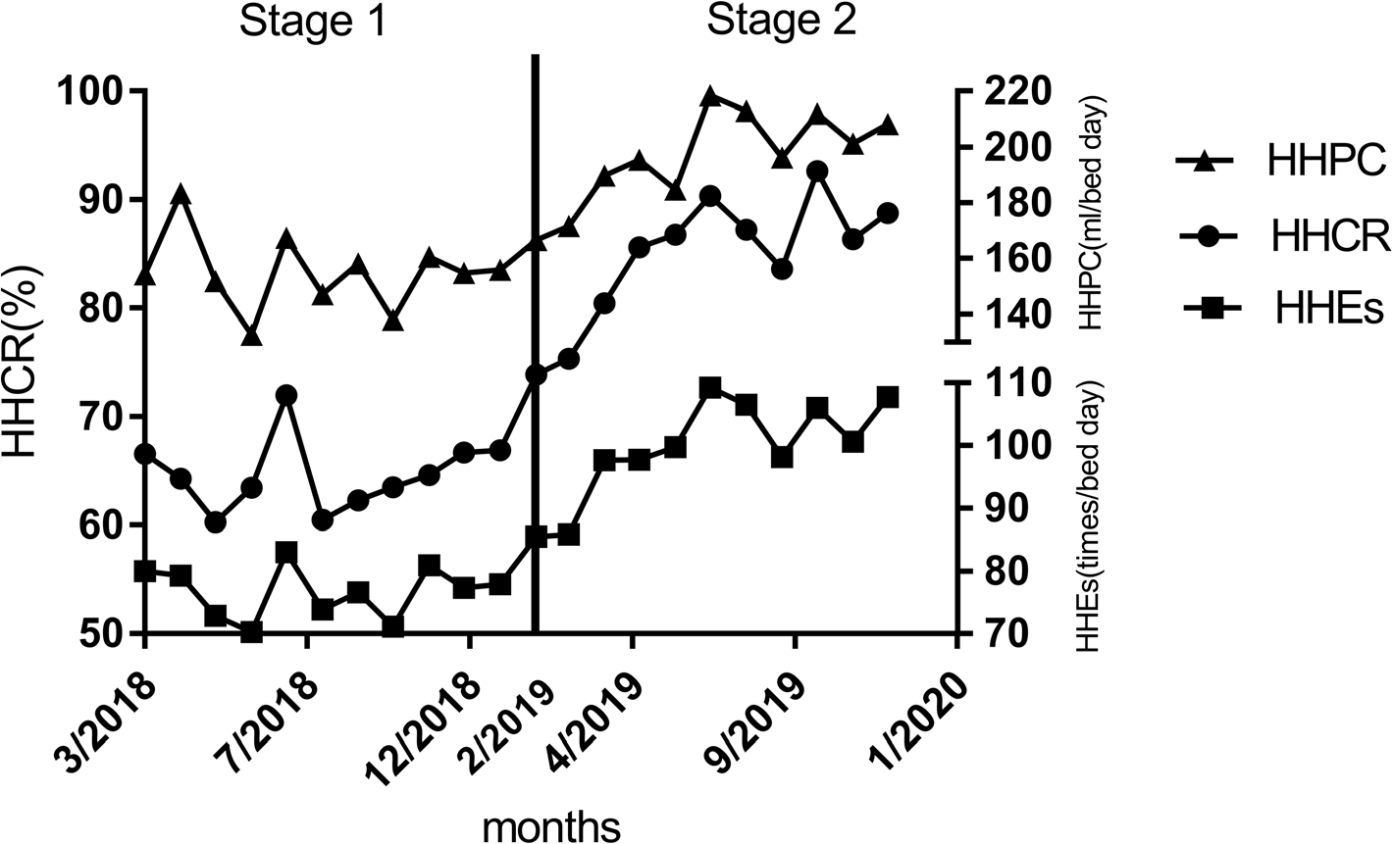
The variation of hand hygiene compliance rate, hand hygiene events per bed day, and hand hygiene product consumption per bed day in stages 1 and 2. The black spots line shows the hand hygiene compliance rate, the triangle spots line shows the hand hygiene product consumption per bed day, and the square spots line shows the hand hygiene events in stages 1 and 2. The solid vertical line depicts the application of the type2 EHHMS (Feb. 2019). The vertical lines divide the time into two stages, which are stage 1 and stage 2. Note. HHEs, hand hygiene events; HHCR, hand hygiene compliance rate; HHPC, hand hygiene product consumption

In February 2019, when the type2 system was introduced, the HHEs per bed day and the consumption of ABHRs & liquid soaps per bed day all increased immediately by 13.565 times/bed day and 26.286 ml/ bed day, respectively. Afterward, they increased by 1.829 times/bed day and 4.571 ml/ bed day over time, respectively, as shown in Table 2-3 and Fig. 3, 4, 5.

**Table 2:** The variation of hand hygiene events per bed day in stages 1 and 2

HHEs	Coef.	Ne Wey-West Std. Err.	*t*	*P* > *|t|*	[95% CI]	
*_t*	.015	.331	0.04	0.965	−.681	.710
_x2019m2	13.565	3.940	3.44	0.003	5.287	21.843
_x_t2019m2	1.829	.591	3.09	0.006	.587	3.071
_cons	76.565	2.368	32.33	0.000	71.589	81.540

Note. *HHEs* hand hygiene events, *Coef* coefficient, *Std. Err.* standard error, *CI* confidence interval, _*t* time since the start of the study, *m* month, *_x(trperiod)* dummy variable which representing the intervention periods (preintervention periods 0, otherwise 1), *_x_t(trperiod)* the interaction of _x and _*t*, *_cons* constant

**Table 3:** Changes in consumption of ABHR and liquid soap per bed day in stages 1 and 2

HHPC	Coef.	Ne Wey-West Std. Err.	*t*	*P* > *|t|*	[95% CI]	
*_t*	−.721	1.094	−0.66	0.518	−3.019	1.577
_x2019m2	26.285	8.470	3.10	0.006	8.490	44.079
_x_*t*2019m2	4.571	1.446	3.16	0.005	1.533	7.609
_cons	158.496	8.127	19.50	0.000	141.420	175.571

Note. *HHPC* hand hygiene product consumption, *Coef* coefficient, *Std. Err.* standard error, *CI* confidence interval, _*t* time since the start of the study, *m* month, *_x(trperiod)*, dummy variable which representing the intervention periods (preintervention periods 0, otherwise 1), *_x_t(trperiod)*, the interaction of _x and _*t*; _cons, constant

Spearman correlation test showed that the HH compliance rate from direct observation was correlated with HHEs per bed day (*r* = 0.956, *P* < 0.001) and consumption of ABHRs & liquid soaps per bed day (*r* = 0.904, *P* < 0.001), respectively. In the third stage, the HH compliance rate of 1, 4, and 5 of “the WHO’s five HH moments” detected by the EHHMS was 77.75% (239,494/308019), which was lower than 89.16% (1112/1247) observed by the direct observation method, and the difference was statistically significant.

## Discussion

HH promotion is an essential part of HAIs control. The strategies to promote HH in the guidelines issued by WHO included changing the medical institutions’ cultural environment, providing accessible HH products, training and education of HCWs, workplace reminder signs, and HH monitoring & feedback [4]. Monitoring & feedback is one of the essential and standard methods in the multi-mode promotion strategy of HH. The purpose of monitoring is to obtain the status of HH compliance of HCWs and assess interventions’ effectiveness. However, only monitoring does not improve HH compliance among HCWs; it must complement HH data’s feedback. In the three stages of this study, the monitor methods and the contents of the feedback were different. However, the frequencies of feedback were identical, which all were monthly feedback. To remove the original trend of the outcome variables before the interventions, we applied the ITS analysis method.

The research design of ITS is regarded as the most robust quasi-experimental design. Because the design fully considered and balanced the outcome variables’ variation trend before the intervention, relatively stable results could be obtained even if no control groups were set. According to the literature [18, 19], the HH compliance rate trend is often seasonal and self-correlation. The conventional statistical methods can not be suitable for analysis, and the application of ITS analysis can effectively solve these problems. In the first stage, the direct observation method was adopted for monitoring, and the monitoring results were fed back monthly. The HH compliance rate of HCWs showed an increasing trend of 0.250% per month. When the type1 EHHMS was introduced in March 2018, there was no significant increase in the HH compliance rate trend of HCWs compared to the first stage. The outcome indicated that the type1 EHHMS had no significant difference with the direct observation method in promoting the HCWs’ HH. The reason may be that HH’s feedback method was not significantly different from the first stage, both of which were monthly feedback. However, the HH compliance rate of HCWs in the first month using the type2 EHHMs increased by 13.324% immediately and increased by 1.242% over time. Although the HH data were fed back monthly in the third stage, the type2 EHHMS can immediately remind HCWs to carry out HH. The monitoring result indicated that this real-time reminder was beneficial to improve the HH of HCWs. It had been reported that EHHMS with real-time reminder and feedback function could promote the HH of HCWs [12, 20–22], which is consistent with the results of this study.

In this study, some advantages of EHHMS over the direct observation method were found: 1. It is more comprehensive and can monitor the HH around the clock [23, 24]. In the second and third stages, the direct observation method only observed 3133 HHEs, while the number of HHEs monitored by the EHHMS was 425,602, which was much higher than the direct observation method. 2. The type2 EHHMS has real-time reminders and feedback functions, which can better improve the HH compliance of HCWs. 3. The EHHMS can reduce the influence of the Hawthorne effect. In the third stage, the HH compliance rate (before and after contact with the patient, after contact with the patient’s surroundings) monitored by the type2 EHHMS was far lower than the rate observed. One of the primary reasons may be that the Hawthorne effect of EHHMS was different from direct observation [13]. Although with the EHHMS, HCWs would feel monitored at the beginning because of wearing identity badges. However, as time went by, HCWs would get used to wearing identity badges, and the Hawthorne effect would gradually decrease. Vaisman A et al. [25] found that the number of HHEs recorded by EHHMS was 2.5 times higher when the observer was present than absent, and the difference may be attributed to the Hawthorne effect. Besides, the EHHMS was not as accurate as of the direct observation method in the identification of HH indicators [26], so more HH moments were recorded [27].

However, EHHMS also has some limitations [12, 16, 26], such as the inability to monitor the correctness of HH, which usually costs a lot, and the failure to follow the five HH moments recommended by WHO adequately. The type2 system in this study, like many systems [24, 28], can only detect 1, 4, and 5 of the five WHO’s HH moments, but not 2 and 3. However, it has been reported that it can also be used as an alternative to HH compliance monitoring [29, 30], which can detect 80–85% of HH indicators [31, 32].

The EHHMS used in this study can monitor the number of HHEs and the consumption of ABHRs & liquid soaps per bed day. At present, many EHHMSs have this function [24, 28]. Although it can not directly calculate the HH compliance rate, it can also reflect the HH compliance of HCWs from one side. In this study, the ITS analysis was performed to analyze the number of HHEs and HH product’s consumption in the second and third stages. The findings were consistent with the HH compliance rate trend of HCWs obtained by direct observation. Furthermore, the correlation analysis results also showed that the HH compliance rate obtained by direct observation was positively correlated with the HHEs per bed day and HH product’s consumption per bed day obtained by EHHMS. It was suggesting that the results of EHHMS could be used to replace the direct observation method to evaluate the HH compliance of HCWs.

### Study limitations

There are some limitations to this study: 1. ITS design belongs to a quasi-experimental study. Although the research variables’ trend was controlled before the interventions, other confounding factors affecting the change of research variables could not be considered. 2. It has been reported that the compliance of HCWs wearing identification badges is one of the obstacles to the use of EHHMS [33]. However, in this study, supervisors were set up to urge HCWs to wear identification badges. Therefore, compliance with the spontaneous application of identification badges by HCWs could not be monitored.

## Conclusions

In summary, the number of HHEs and consumption of HH products monitored by the EHHMS in ICU can well reflect the HH compliance of HCWs. HH compliance of HCWs can be improved by HH monitoring and feedback. The EHHMS, with instant reminder and feedback function, can promote HH more obviously. At present, the EHHMS cannot adequately detect the WHO’s five HH moments. A more intelligent and accurate EHHMS, such as using the camera with artificial intelligence image recognition to identify the HCW’s HH moments and HHEs, is worth our expectations.

## Supplementary Information

**Additional file 1.** The technical parameters. Part 1. The type1 EHHMS equipment technical parameters. Part 2. The type2 EHHMS equipment technical parameters.

## Data Availability

The data used in the study was available from the department of nosocomial infection control of the Shenzhen Hospital of the University of Chinese Academic of Science.

## Abbreviations

EHHMSs: Electronic hand hygiene monitoring systems
HH: Hand hygiene
HCWs: Healthcare workers
ICU: Intensive care unit
ITS: Interrupted time series
HHCR: Hand hygiene Compliance rate
HHEs: Hand hygiene events
HAI: Healthcare-associated infection
ABHRs: Alcohol-based hand rubs
Coef: Coefficient
Std. Err.: Standard error
CI: Confidence interval
m: Month
