# Supplementary material for: Comparison of two electronic hand hygiene monitoring systems in promoting hand hygiene of healthcare workers in the intensive care unit
Source: BMC Infect Dis. 2021 Jan 11;21:50. doi: 10.1186/s12879-020-05748-3 (PMC7802277; doi:10.1186/s12879-020-05748-3)
Supplement: Supplementary file 1 — Additional file 1. The technical parameters. Part 1. The type1 EHHMS equipment technical parameters. Part 2. The type2 EHHMS equipment technical parameters. [file 12879_2020_5748_MOESM1_ESM.docx]

**Part 1：The type1 EHHMS equipment technical parameters.**

**1. Automatic induction reservoir**

A. Rechargeable lithium battery, a charge can be used for 2-3 months.

B. Operating current: Standby ≤20uA, RFD-like transmitting current ≤300mA.

C. RF receiving sensitivity: when transmitting signal is -100dbm, bit error rate ≤10%.

D. 433M transmitting power: 10dBM.

E. Identification distance: 0.6m.

**2. RFID badge**

A. Wide range of identification, within a radius of 60cm.

B. Recognition speed is fast, with anti - rewrite function.

C. Storage capacity: 40-60 pieces of hand hygiene information.

**3. Acquisition and display all-in-one machine**

A. Automatic collection of recorded data.

B. Wide range of identification, within a radius of 60cm.

C. Display hand hygiene information of medical staff, hand hygiene ranking, etc.

**Part 2：The type2 EHHMS equipment technical parameters.**

**1. Badge**A. Lithium power supply, low voltage alarm function.

B. Operating current: Standby ≤60uA, operating current ≤5mA, RF transmitting current ≤30mA.
C. 433M receiving sensitivity: when the transmitting signal is -100dbm, bit error rate ≤10%.

D. RF transmission power: 10dBM.
E. The badge can stand standby for 24 months and charge for 30 days.
F. The response time to the recognizer is less than 100 milliseconds.
G. Compatible with existing hospital badge, divided into vertical version and horizontal version.
H. With the function of sound and light, it can remind and guide users to conduct hand hygiene if they fail to wash their hands at the time of hand hygiene.

**2. Bed area recognizer**
A. Lithium power supply, low voltage alarm function.

B. Operating current: Standby ≤20uA, RFD-like transmitting current ≤300mA.

C. 433M receiving sensitivity: when the transmitting signal is -100dbm, bit error rate ≤10%.

D. RF transmission power: 10dBM.
E. Recognition distance: 0.5-2.0 m adjustable.

F. Working time of a single charge is more than 12 months.
**3. Liquid bottle recognizer**

A. Lithium power supply, low voltage alarm when the voltage reaches 3.3V.
B. Operating current: Standby ≤20uA, RFD-like transmitting current ≤300mA.
C. RF receiving sensitivity: when transmitting signal is -100dbm, bit error rate ≤10%.
D. 433M transmitting power: 10dBM.
E. Identification distance: 0.6m.
F. It can stand on standby for 2 years and can be used for 6 months if it is pressed 200 times a day.
**5. AP**
A. Supply voltage: 12V power adapter for direct supply.

B. Operating current: normal ≤100mA, RF emission ≤35mA.

C. 433M receiving sensitivity: when the transmitting signal is -100dbm, bit error rate ≤10%.

D. 433M transmitting power: 10dBM.
E. Disconnect the network to store 1000 violation information.
F. Use mobile 4G network transmission, do not use the hospital network.
**7. Entry and exit monitor**
A. Supply voltage: 12V power adapter for direct supply.

B. Operating current: normal ≤20mA, RFID-like transmitting current ≤300mA.
C. Recognition distance: adjustable from 1.0 m to 2.0 m.
D. Distinguish between entrances and exits.
